# A dual layer secure and energy-efficient model for border surveillance using sea lion inspired strategy in wireless sensor networks

**DOI:** 10.1038/s41598-025-07999-z

**Published:** 2025-07-10

**Authors:** Jayachandran J, Vimaladevi K

**Affiliations:** School of Computer Science Engineering, Vellore Institute of Technology, Vellore, 632014 Tamil Nadu India

**Keywords:** Wireless sensor networks, Clustering, Data aggregation, Routing, Sea lion optimization, Security, Energy efficiency zone optimization, Mathematics and computing, Computer science, Electrical and electronic engineering

## Abstract

In recent years, networks of sensors have gained significant attention for security-sensitive applications, such as border monitoring, where network durability, efficiency, and security are of utmost importance. In sensor networks, security related measure will often lead to an increase in energy consumption, hence, designing efficient energy conserving protocols with robust data aggregation methods are crucial in line with better duty-cycling idle nodes in the network. This paper presents a Dual Layer Sea Lion Optimization algorithm (DL-SLnOA) model, a bio-inspired, clustering and routing that focuses on energy efficiency and security to enhance performance under challenging conditions. The proposed DL-SLnOA combines the SLnO algorithm with a dual-layer security framework to optimize the selection of Cluster Heads (CHs) by using adaptive exploration and exploitation methods, which dynamically position CHs to balance energy consumption, proximity, and trustworthiness. DL-SLnOA first layer incorporates dynamic trust score update to ensure only reliable nodes participate in communication and second layer implements anomaly detection methods, which are used to identify malicious behaviour with inherent high detection rates and least false positive rates. Unlike other methods, this solution tends to firmly segregate legitimate from malicious nodes, thereby reinforcing the network. The simulation results for selective forwarding attacks with 10 malicious nodes in 100 iterations detected threats within 7 rounds with a packet transmission efficiency of 97.9%, while for wormhole attacks detection within 2 rounds, packet transmission efficiency of 98.3%, and average energy consumption of about 0.122J, significantly increasing the network lifetime. These improvements that provide an energy-efficient and secure platform strongly reinforce the network against security threats by further extending the life span of the network, making DL-SLnOA a scalable solution.

## Introduction

Wireless sensor networks (WSN) have evolved into a core technology in various domains such as environmental monitoring, smart city designs, military applications, and border protection. In applications that are prone to security breaches, for example in border monitoring, WSNs provide tactical aid for active surveillance and information gathering which is critical since information must be precise and timely for decisions to be made^1,2^. Border surveillance holds a crucial responsibility in holding peace and protection of country citizens with round the monitoring of hundreds of kilometres including extreme terrain and unpredictable weather conditions. Monitoring is mostly done through manual patrol workforces and military across the borders, yet the required resources and workforce commitment is ever increasing due to terrorist’s invasions, illegal movement of immigrants and more^3^. Energy consumption is one of the serious challenges experienced by WSNs whilst performing border monitoring; this is because WSN-powered sensor nodes are usually positioned in concealed locations where routine battery maintenance is impractical . Data transmission is achieved by selecting the most energy-efficient path, thereby minimizing long-distance communication and ensuring balanced energy consumption across the network^4,5^. Equal energy depletion among the CH and its Cluster Members (CM) are vital to ensure effective inter and intra-cluster communication by preventing nodes from early failure i.e. CH might be overloaded since it is located near to the Base Station(BS) and CM might use more energy where distance to CH is high. Such scenarios might lead to premature node failures, coverage gaps and network disconnectivity which will affect the overall network performance. In addition, safeguarding the information transmitted from CH to BS is crucial to ensure data reliability and integrity with a robust data encryption mechanism^6^. Conventional approaches for minimizing energy expenditure tend to be clustering-oriented, in which some nodes are designated as CH for the primary purpose of data collection and forwarding to BS. However, without dynamic optimization, a continuous and independent data transmission between CM and CH, and CH to BS considerably increases the energy depletion of nodes and hastens node mortality. Allocating an appropriate CH with right position to its CM will reduce the uncertainties in optimization decision making using meta-heuristic techniques. Since meta-heuristic approaches deal the CH selection and routing path determination as an optimization problem with a specific objective function and with continuous iteration an optimal solution will be identified using algorithms like cat swam, fish migration, ant colony optimization, and genetic algorithm^7^. Conventional clustering protocols like LEACH can guarantee optimal CH selection but fails to concentrate on secure communication even though random integer technique used to rotate CH for each iteration. Thus, block chain and Real Time Message Content Validation (RMVC) was integrated with LEACH was proposed to detect malicious node with WSN network using secure hash algorithm. But, the computation overhead and the complexity of cryptographic functions have imposed some challenges in making it into practice. To tackle this issue, improved threshold value was adapted for optimal CH selection and trust based routing model was proposed to ensure the trustworthiness of route reliability using direct and indirect trust opinion values for resisting routing attacks^8^. Secure communication can be achieved through trust score evaluation techniques, which help in establishing secure routing paths for data transmission. Trust is typically used to evaluate the reliability of nodes, while network security also involves additional measures such as intrusion detection and firewalls, misbehaviours like packet loss and data rearrangement are examples of security issues that can occur in WSNs^9^.

**Figure 1:**
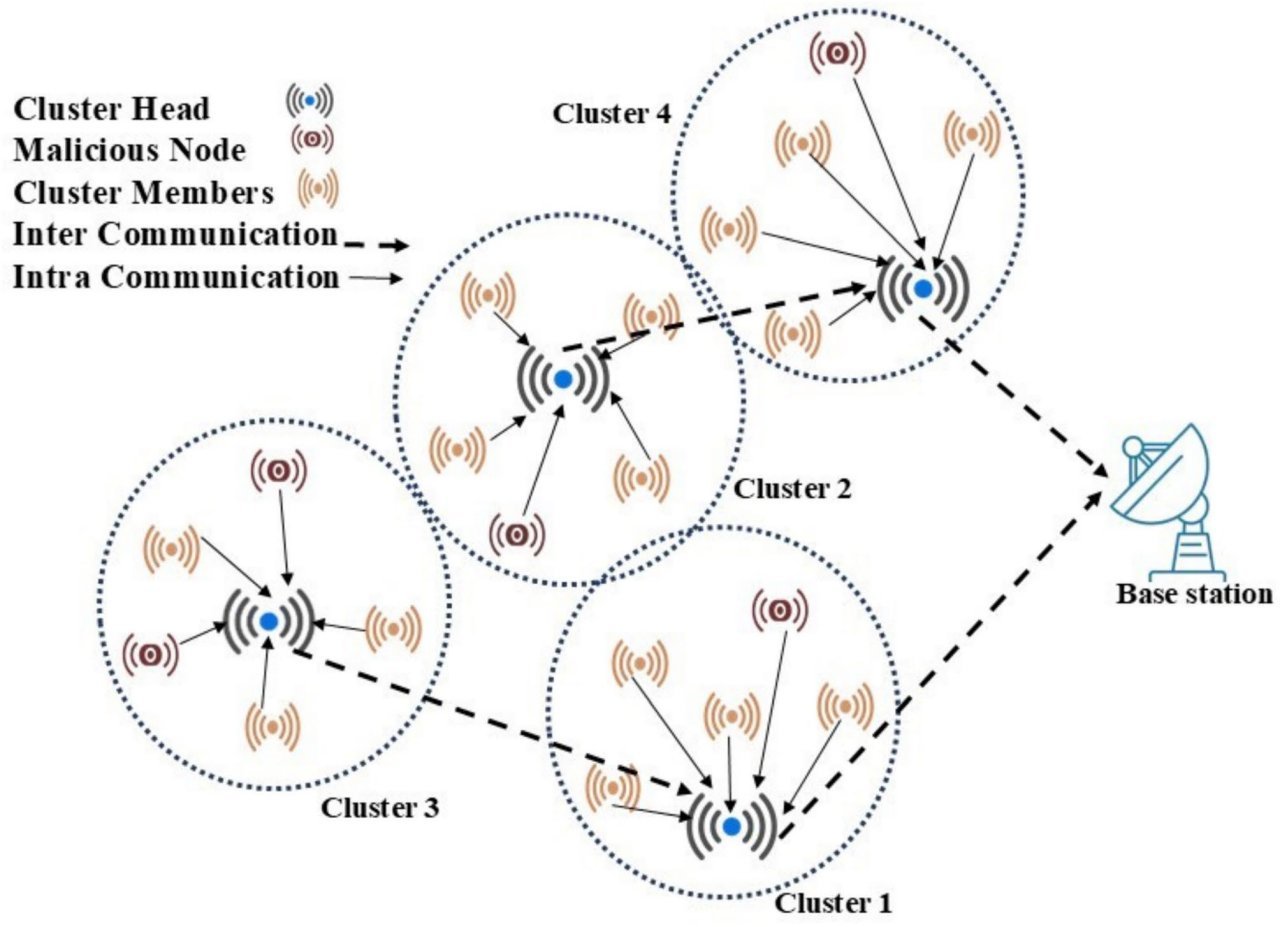
Network diagram of the proposed DL-SLnOA model.

This initiative is prompted by the rising demand for reliable, energy-efficient, and secure WSN in critical applications, especially in border surveillance and other high-risk environments. In these applications, WSNs are frequently set up over large isolated, and occasionally hazardous terrains with limited energy resources and little maintenance. Therefore, it is evident that adaptive clustering and routing systems that optimize energy consumption and include robust security mechanisms to safeguard critical data are required, because CH is essential for managing data flow within clusters, determining the optimal CH locations and rotation schedules are essential to lowering energy usage and increasing network lifetime. Additionally considering the increasing importance of data integrity and secure communication, the model must incorporate security layers that can spot anomalies and maintain a high degree of confidence in network connections. In order to provide resilience and longevity in security-sensitive applications, the objective of this work is to address premature convergence issue of WSN along with optimal CH selection and its position with reduced energy consumption, increase network longevity and secure data transmission using bio-inspired algorithms need to be developed and implemented. This research study aimed to address and overcome the above challenges with dual-layer secure routing using Sea-Lion algorithm and cluster-based network division of WSN. This article presents a unique clustering and routing framework that incorporates the SLnO method for effective CH rotation and selection. The SLnO algorithm is selected over other bio-inspired algorithms due to its effectiveness in node deployment, coverage rate and its flexibility towards exploration and exploitation features, and it also achieve effective utilization of network resources. The SLnO algorithm optimizes CH placements by balancing node trustworthiness, communication distance, and energy load through exploration and exploitation stages. This DL-SLnOA dual-layered security method, which also includes behavior anomaly detection and trust score updates, enhances network resilience by promptly identifying and hindering any threats by focusing on both energy efficiency and security, this strategy aims to improve network durability while providing robust defense against security threats, making it a feasible choice for long-term, high-security in WSN installations. Figure  1 illustrates the network architecture of the proposed DL-SLnOA model, primary contributions are as follows:

1. SLnO guarantees the best CH choice, maintaining efficient coverage and connectivity while improving energy efficiency.
2. The proposed DL-SLnOA model successfully detects compromised nodes by fusing trust scores and behaviour-based anomaly detection averting possible security lapses in data transfer.
3. According to a temporal complexity analysis, this model is appropriate for large-scale border monitoring applications since it scales well with network size. Through comparative analysis and simulation, our model outperforms conventional methods on measures such as data integrity, detection accuracy, and average energy consumption.

This paper is structured as follows: Section “Related work” provides a review of related studies on trust-based, secure, and energy-efficient communication methods in WSNs. Section “Proposed system model” introduces the proposed DL-SLnOA approach, highlighting its CH selection, clustering-based secure communication, and routing mechanisms. Section “Simulation setup” details simulation parameters and metrics for trust-based malicious node detection speed, packet transmission, and network utilization. Finally, “Conclusion and opportunities of future research” concludes the study, summarizing the proposed DL-SLnOA approach effectiveness in improving network security and energy efficiency, and outlines the potential future directions.

## Related work

This segment reviews existing research on secure and energy-efficient routing in WSNs, essential in various critical applications. A major challenge in WSNs is achieving a balance between conserving energy and maintaining secure communication, which extends network lifespan and safeguards against malicious threats. Researchers have employed multiple strategies, such as clustering methods, trust-based routing, and machine learning models, each contributing uniquely to improving network security and efficiency. This section outlines significant advancements and limitations in recent methods, by providing a foundation for the enhancements proposed in this research work. In  ^10^ author suggested a trust-based secure and energy-efficient routing protocol (TBSEER) designed to address security and utilized energy issues, combines dynamic direct trust, derived trust, and energy dependent trust values to resist common attacks like black hole, sinkhole, hello flood, selective forwarding (SFA), and wormhole attacks (WHA). This protocol incorporates a flexible penalty strategy and volatility factor to enhance trust assessment accuracy and accelerate the detection of intrusive nodes with Sink Node (SN) which calculates the comprehensive trust value according to the trust scores communicated by nodes, TBSEER reduces communication congestion and energy consumption. It also actively avoids wormhole assaults by choosing safe and energy-efficient pathways via a multi-path strategy, in comparison to conventional trust-based methods, the results demonstrate that TBSEER enhances network stability, lowers energy consumption, and expedites the discovery of malicious nodes. The author^11^ proposed an Efficient Trust-based Energy-aware Routing Scheme (ETERS), which is a secure protocol that defends against malicious activities such as sybil, black hole, and grey hole attacks using a flexible beta distribution algorithm that enables quicker trust recovery in the event of an attack, ETERS determines node trust based on communication, energy, and data reliability. This Enhanced Cluster Head Selection Algorithm (ECHSA) method rotates CHs regularly according to energy and distance, balancing the load, Node IDs, location, and a variety of trust measures are used by a trust-driven intrusion detection algorithm to evaluate node credibility. It uses AODV with thorough path cost metrics to guarantee reliable routes with low latency and high residual energy. MATLAB simulations verify this, demonstrating notable gains in throughput, packet delivery, and energy efficiency in in clustered WSNs. In^12^ author proposed a Temperature-Aware Trusted Routing Scheme (TTRS), robust multifactor routing protocol that prioritizes security and energy efficiency by evaluating nodes based on trust scores, energy levels, and hop counts. TTRS establishes secure and energy-efficient routes by selecting trustworthy nodes and incorporating an algorithm for detecting hotspot nodes to identify malignant nodes in harsh environments using a beta distribution-based trust model, TTRS ensures rapid trust recovery under attack. The scheme integrates direct, derived, feedback, and energy trust to calculate reliable trust values, enhancing data integrity. MATLAB-based evaluations reveal superior performance over other protocols in packet transmission, lifespan, and power conservation, making it highly suitable for environments with malicious threats. In  ^13^ author introduced a Taylor-based Cat Salp Swarm Algorithm (TaylorC-SSA) is a novel power-efficient hop-based routing method, which addresses high energy consumption of battery-powered sensor nodes, combines the Cat and Salp swarm algorithm with Taylor series to select optimal hops for data transmission. Taylor C-SSA operates in two stages: selecting energy-efficient CHs using LEACH protocol and securely transmitting data across optimal multi-hop paths. A trust model enhances routing security, incorporating direct trust, derived trust, integrity, and data forwarding rate into the objective function. Experimental findings highlight superior performance in terms of energy efficiency, delay, throughput, and node longevity, making it effective for WSNs in energy-sensitive applications. In  ^14^ author introduces an Evolving Trust-based Dynamic Model for Autonomous (ETDMA), aimed at enhancing the security and reliability in dynamic environments. ETDMA calculates trust using novel trust features: geographical, joint work relationship, collaboration frequency duration, and reward, integrated with direct and indirect trust. Experimental results indicate ETDMA’s superior performance, achieving a 94% malicious node detection rate, 92% accuracy, and a low false negative rate, outperforming RBC-IDS across various metrics. In  ^15^ author presents an improved Honey Badger Algorithm (I-HBA) using refined hop count routing protocol to address energy efficiency and security. I-HBA follows two stages: selecting energy-efficient CHs and establishing optimal multi-hop paths to BS. It uses a multi-target fitness function with eight parameters, optimizing energy usage and enhancing secure routing, unifying direct and indirect trust, data accuracy, and forwarding speed, further securing data transmission. In relation to other protocols I-HBA-based approach significantly improves data packet delivery and routing efficiency, showcasing its potential to enhance performance, scalability, and security in critical applications. In  ^16^ author introduces a Competitive Verse Flower Pollination (CVFP) algorithm for secure routing protocol which optimizes routing by incorporating factors such as power consumption, range, response time, and reliability. Link longevity is used to select the most efficient path, nodes have high energy are chosen as CH to extend network lifespan and reduce delays. simulation results show the proposed algorithm achieves low delay (0.204 s), high residual energy (0.093 J), a peak transmission rate of 94.59%, and a reliability value of 0.713, proving its effectiveness for secure, energy-efficient IoT routing. This study^17^ proposes an Energy Efficient Rider Remora Routing (EERRR) protocol to enhance network lifetime and secure communication. CHs are selected using a fuzzy logic-enhanced balanced cost approach to optimize data transmission. The EERRR protocol used to find the best path based on residual energy, distance, and node centrality, simulation results show improved packet delivery, energy efficiency, throughput, and network lifespan. Future work will integrate machine learning and cryptographic techniques to further improve security and efficiency. In  ^18^ the author suggested a Secure Energy-Aware Meta-Heuristic Routing (SEAMHR) protocol aims to improve network security and performance which utilizes for intelligent learning and makes routing decisions relies on hop counts, link integrity, and residual power, and integrates counter encryption and autoencoding model for secure data encryption and authentication which enhances energy efficiency, network throughput, packet delivery ratio, and route maintenance, outperforming existing protocols like SEHR, SecTrust-RPL, and HBEER, results show significant energy savings and improved data transmission. In  ^19^ author suggested Trust Aware Oppositional Sine Cosine-based Multihop Routing (TAOSC-MHR) is designed to improve the resilience through trust and energy optimization. It starts with a weighted cluster formation scheme to organize the network and select CHs. The proposed protocol employs oppositional learning in conjunction with sine cosine optimization for optimal route determination. This objective function incorporates remaining power, node separation, and trust ratings, extensive simulations demonstrate that the TAOSC-MHR protocol outperforms existing techniques in terms of survivability and performance. In  ^20^ author proposed a dual-energy and secure layer routing approach (DESLR) to enhance optimal energy use and signal reliability which introduces through a channel-based trust model, evaluating direct and derived trust metrics to detect malignant nodes while accounting for the impact of unreliable acoustic channels. This method uses an enhanced ant colony to opt for low-latency, optimal power routing paths through a layered clustering approach is applied, and a dual tier fuzzy mechanism is used to select CHs for balanced power utilization, results demonstrate DESLR effectively detects malignant nodes, reduces power utilization, and outperforms other protocols in terms of delay and packet loss, this approach significantly improves the survivability and reliability of acoustic networks^21^. This paper presents a lightweight secure routing (LSR) approach by addressing multi-objective optimization, which includes mitigating the energy-hole problem, enhancing coverage, and improving security, energy efficiency, and QoS. LSR algorithm incorporates four models: a hybrid deployment model, dynamic connectivity, adaptive QoS, and adaptive security which uses Ant Colony Optimization (ACO) to select routing paths, integrates direct and indirect trust calculations for security, and improves network connectivity to address low node density issues. Simulation results show that LSR outperforms existing methods in terms of power drawn, network longevity, and packet success rate and routing delay. Future suggestions for extending the algorithm to different network shapes, enhancing indirect trust calculation, and integrating blockchain for improved data security^22^. This paper introduces the QoS-aware energy balancing secure routing (QEBSR) algorithm addressing the critical challenges of energy consumption, QoS, and security. Proposed algorithm uses an ant colony approach to optimize routing paths while considering both energy efficiency and security. Improved heuristics are Suggested for computing latency and trust values along routing path, resulting in better network performance, results demonstrate QEBSR outperforms existing algorithms in terms of lifespan, latency, and data forwarding through trusted nodes. QEBSR algorithm also employs weight vectors to balance objectives, though future work may explore solutions without relying on them. In  ^23^ author presents the MAC Centralized Routing Protocol (MCRP) designed to minimize utilization of power and detect wormhole attacks, employs a high-power BS to manage energy-demanding tasks like routing path calculation and network topology monitoring, reducing the load on sensor nodes. MCRP protocol Relies on a time threshold limit to detect wormhole attacks by comparing expected and actual transmission times. Simulation results show that MCRP improves scalability, power utilization, latency, throughput, and frame reception ratio. MCRP’s performance enhances as the number of nodes increases, making it effective in large-scale WSN deployments. In  ^24^ author introduces a Dual-Tier Cluster-Based Routing (DTC-BR), for mobile sensors to address challenges like power utilization, connectivity, scalability, and security which divides the network into virtual zones, covering the main connectivity zone and the potential cluster zone, using a dual-tier clustering mechanism which selects the most suitable sensor node as a CH based on distance and connectivity, It has been observed that DTC-BR improves lifespan by 6% to 37% compared to existing protocols like DDR, MCCA, LEACH-MEEC, and LEACH-M. Author^25^ presents Crossover Mutated Marriage in Honey Bee (CM-MH) a redesigned secure routing solution which combines path optimization and cryptographic model for enhanced security, to select optimal paths for secure transmission between source and destination. CM-MH approach outperforms existing models like PSO, FF, GA, and MHBO in terms of distance and trust values, with improvements of up to 15.35%. In article  ^26^ author proposed a joint power allocation and secure routing strategy (JPASR) for critical event reporting includes uplink transmission, which maximizes secure connection probability while minimizing power consumption and for downlink transmission, power-oriented multi-point relay selection method to select energy-efficient relays for message broadcasting, and prolonging lifespan. A multi-level sleep scheduling strategy is also employed to save energy by reducing retransmissions and optimizing sleep cycles, extensive simulations show that the method outperforms existing approaches in terms of lifespan, utilization of power, and security. Reference^27^ proposed a cluster-tree-based trusted routing method (CTTRG), which integrates a time-sensitive trust to analyze sensor node behaviour using criteria such as blackhole, sinkhole, grayhole, wormhole, and flooding probabilities. Routing utilizes the Grasshopper algorithm to construct secure communication paths via a trusted routing tree (GTRT) a multi fitness function is designed to evaluate the distance, trust level, and power of CH, results demonstrate that CTTRG improves detection speed, reduces data loss rate, and minimizes e2e latency compared to other protocols like TBSEER, TSSRM, and TESRP. In  ^28^ author introduces a novel relay ring (ReRR) routing to enhance anonymity of source location unlike existing fake packet-based protocols, which provide short-term protection, ReRR guarantees long-term Source location protection with an energy-efficient routing algorithm, results show that ReRR outperforms traditional protocols like DissRand and DistrR in terms of location reliability, power utilization, and lifespan. ReRR achieves high path diversity and adversary obfuscation, ensuring improved long-term privacy and provides multiple relay nodes, safeguarding the source location and ensuring reliable protection. The author^29^ presents a new fuzzy-based secure clustering algorithm to enhance energy efficiency and security uses trust-driven fuzzy to identify malignant nodes and select reliable paths for transmission, improving both network security and performance, results demonstrate proposed method reduces power utilization, enhances packet delivery, and decreases data transmission delay, leading to improved network lifetime. Reference^30^ introduces a Trust Aware Clustering Technique based on an Improved Rat Swarm Optimizer (TACTIRSO). TACTIRSO selects CHs securely by considering nodes trust values and residual energy. Proposed approach uses an enhanced Rat Swarm Optimizer with energy and trust-aware initialization and local search strategies, a new selection function, incorporating leftover power and trust values to select reliable CHs. This exponential sliding average model dynamically adjusts threshold values based on network conditions and results show TACTIRSO outperforms existing methods in terms of power efficiency, trustworthiness, and network stability. In  ^31^ author proposed a new Trust-Based Secure Intelligent Opportunistic Routing Protocol (TBSIOP) to enhance security and efficiency in data packet transmission. This protocol computes a node’s trust factor using three attributes: sincerity in forwarding data packets, sincerity in acknowledgments (ACKs), and energy depletion, these factors are used to identify and prevent malicious nodes from participating in the routing process. TBSIOP is integrated with the Intelligent Opportunistic Routing Protocol (IOP) to improve WSN security and performance. Simulation results show that TBSIOP outperforms existing protocols in terms of packet delivery ratio, energy consumption, end-to-end delay, network lifetime, and average risk level, this protocol is less susceptible to grey and black-hole attacks, ensuring secure routing. TBSIOP reduces the average risk level by 2.87% and increases the packet delivery ratio by 3.06% compared to IOP and other protocols like SQEER, it significantly lowers the end-to-end delay and enhances network lifetime with lower energy consumption. Future work includes exploring additional WSN attributes for trust computation and developing robust protocols to tackle more critical security threats. In  ^32^ author presents Enhanced Multi-Attribute-Based Trusted Attack Resistance (EMBTR) algorithm for secure routing, leverages QoS parameters like stability index, reliability factor, and elapsed duration, for enhancement of network security and performance. EMBTR isolates misbehaving nodes based on trust metrics, ensuring reliable communication routes and combating trust-related attacks, such as selective packet forwarding, results show that EMBTR outperforms existing models like TSRM and TARF in terms of energy optimization, rate of detection, and data transfer rate. This approach minimizes false positives and improves network security while maintaining lower energy consumption. In  ^33^ author proposed a Multidimensional Secure Clustered Routing (MSCR) scheme based on binomial distribution to defend against internal attacks, incorporating distance, energy, security, environment domains, to balance security, transmission performance, and energy efficiency. It prevents malicious nodes from becoming cluster heads, improving network security, enhances energy efficiency, prolongs network lifetime, and strengthens security by incorporating trust values into CH election. Simulation results show that MSCR performs well on power consumption and lifespan. Future work will explore the role of different distributions in secure routing in hierarchical WSNs. In  ^34^ author presents a cluster-based trusted routing protocol (CTRF), which uses the Fire Hawk Optimizer (FHO) to enhance security and energy efficiency. CTRF method introduces a trust based weighted mechanism that evaluates node trust levels adjusted according to reception rate, redundancy, and energy state based on node behaviours. The FHO-based clustering mechanism selects CH from high-energy, reliable nodes using a cost function considers intra and inter-cluster distances, energy, and cluster size. Trusted routing protocol ensures secure inter-cluster paths by evaluating route energy, quality, reliability, and hops. CTRF improves energy by up to 16.15%, throughput by up to 43.09%, and detection rate by up to 6.26%. Reference^35^ introduces LCASO-MTRM method designed for QoS routing, enhances bandwidth while reducing power usage, latency, and packet loss by incorporating Levy chaos Adaptive Snake Optimization framework. The chaos operator accelerates population diversity and the search process, meanwhile adaptive operator facilitates convergence and prevents stagnation. Proposed algorithm integrates a link trust mechanism, including both direct and indirect trust, to evaluate network performance. Simulation results show that LCASO-MTRM outperforms existing methods, decreasing energy usage by 49.53%, reducing latency by 22.56%, and cutting packet loss by 40.21%, while boosting bandwidth by 6.13% this approach provides superior performance in power efficiency, latency minimization, and packet loss reduction, which is suitable for latency-sensitive real time applications. In  ^36^ author presents the SEHR protocol, to optimize routing decisions, focusing on energy consumption, data security, and route maintenance. It integrates a heuristic function based on factors such as remaining power, hop count, and link integrity to improve network performance. SEHR also employs traffic exploration to prevent link failures and network disconnection. Simulation results show that SEHR enhances network throughput by 18%, reduces packet drop by 42%, lowers energy consumption by 36%, and improves route reliability and network efficiency. SEHR protocol uses a lightweight encryption algorithm to ensure data security. Reference^37^ proposed the SLERP protocol, that combines dynamic trust awareness and load balancing. To predict the dynamic trust degree, enhancing the accuracy and speed of malignant node detection using chebyshev neural network. This protocol establishes an optimal routing path by considering factors like CH trust, power utilized, and lifespan through an analytic hierarchy process (AHP). Genetic based algorithm is employed to optimize the CH selection and routing path, improving efficiency. Simulation results demonstrate SLERP’s ability to reduce power expenditure, improve detection rates, and extend lifespan. SLERP protocol effectively mitigates malicious node attacks while ensuring low energy use. In  ^38^ author suggested Energy Aware and Secure Routing (EASR) for hierarchical cluster-based trust model includes direct, derivative, and comprehensive trust values to identify and thwart routing attacks, EASR improves the LEACH protocol by choosing secure optimal CHs based on trust and energy remaining, this improved protocol enhances both network security and energy efficiency. According to simulation data, EASR performs better than other systems, including TBSEER and MSCR, in terms of packet rate of delivery and power expenditure. In future, machine learning will be used to lower computation overhead and analyze trust values. In^39^, a modified Ant Colony Optimization (ACO) was proposed with unique Trust Evaluation Scheme (TES) which optimizes CH selection and trust score with manifold criterion. In addition, dual-tiered system was introduced to enhance securing of trusted node’s reliability using crowding distance mathematical modelling. The experimentation results were conducted in MATLAB simulation environment and results have shown 35% improvement, 89% reduction, 54% decrease and 73% enhancement on throughput, delay, energy consumption and processing speed performance metrics than approaches like Routing Protocol or Low Power and Lossy Network (RPL), Routing using 6LoWPAN, Secured Routing using Zigbee Cluster Library (ZCL) and Secured routing using Constrained Application Protocol (CoAP). Adapting with ever adding new devices to WSN might impose difficult challenges for security protocols could be the limitation of this work. In^40^, a three layered architecture was introduced with sensor device, edge layer and cloud layers and finally followed by AI model integrated with meta-heuristic algorithm. To ensure maximum secrecy, an Ethereum-based data repositioning framework with trapdoor function was utilized and the conclusive evidence matrix was computed using a simplified consensus module with which an unconventional neural network model along meta-heuristic algorithm to speed up the model. This model was implemented in Python environment with the application interface of NodeJS v.22.x and the initial black chain was designed using Ganache Tool. The results have shown that the proposed model had achieved an improvement of 48.5% on threat detection accuracy and 23.5% reduction in processing time in compared with other existing models considered in this study. In^41^, authors have proposed Sensor as a service (SEaaS) model which is integrated with a decentralized black chain technology to enhance efficiency using simplified public key encryption. An unique black chain based data sharing mechanism was introduced with innovative trading operations for sensed data among diverse stakeholders. The model was implemented in a standard Python environment where the results showed 40% reduction on energy consumption, throughput increase of 18%, latency reduction of 16% and algorithm processing time with 25% reduction than other models like Secure Data Sharing (SDS), Blockchain augmented Data Sharing (BaDS), Anonymized Data Sharing (ADS), and Secure and Light Weight Architecture (SLTA). In^42^, a unique block chain based authentication approach with novel computational security was proposed from different variants of side channel IoT attacks using smart agreement and physically unclonable functions with multiple stages of security implementation. The model environment was created in Python using analytical modelling with lightweight finite field encryption. The results were proved that the proposed model achieved a reduction of 4% processing time, less computation overhead of 5%, enhanced throughput of 1% and 12% less latency and 30% of reduced energy consumption than other models. In^43^, authors introduced a Strategic Game Model (SGM) to computer payoff embrace moves to maximize gain with the help of public and private cloud systems for QoS integration using Global Trust Controller (GTC) and core node selection controller to select appropriate intermediate nodes to forward the data towards BS. An optimal regular IoT node is identified in the final stage to perform routing packets towards the BS through intermediate nodes. The results have achieved 36% of improved accuracy, 25% of reduced energy consumption, 11% faster response time and 27% of reduced cost than other game-based models in terms of security demand and communication demand. In^44^, a multi-agent and multilayered combination of game formulation along with trust model that asses each sensor node to determined the communication participation while transmitting or forwarding data packets from security perspective. The model performance was measured in terms of throughput, delay, intrusion detection rate and false positive rate (FPR). The detection accuracy was improved by 35%, delay was reduced by 13.3%, 59% of improved throughput, and minimum FPR of 0.0127% than other existing models. Yet, AI based approaches suffers with the incapability of adopting more realistic or up-to-data attack data. In recent works, Multi-Agent Reinforcement Learning (MARL) has attained better performance in enhancing the protection and detection efficiency of Intrusion Detection Systems (IDS). But increased network bandwidth consumption and slower training are the challenges faced by this method; to overcome these challenges, Federal Learning (FL) was used to enable decentralized training across network by preserving the privacy of each network participant or resources. In^45^ a novel integrated model named MAF-DRL was proposed which combines the advantages of both MARL and FL to detect WSN based security attacks and threats. This approach encompasses distributed learning across multiple agents for achieving robust detection of threats within the WSN and introduces trust-based scheduling that allocated the required resources dynamically based on the reliability of agents and allow the network to adapt with changing network conditions and node behaviour. The MAF-DRL results were compared with other AI methods like Naive Bayes, Multilayer Perception, Adaptive Boosting and Boosting on attack classification scenarios of Binary Classification Case and Multi-Class Classification case on different attacks normal, black-hole, flooding, gray-scale and scheduling (TDMA) class labels. Table  1 presents a discussion on the existing approaches, objectives and its limitations. From the literature, it is understandable that the need for minimizing the network model limitations with different combination of algorithms and routing protocol mechanisms is evident. The computational complexity of algorithm should not have a great impact of energy consumption since additional energy of sensor node would be consumed for security related model operations like trust based approaches. There still exist a need and opportunity to develop a better combination methods and mechanisms to achieve secure routing of information with minimum energy consumption rate, network over head and reliable routes that transfer information to the control system is widely available by incorporating bio-inspired algorithms.

**Table 1:** Comparison of trust-based methods in WSNs.

Method	Trust metrics	Monitored behaviour	Limitations
TBSEER ^10^	Direct, indirect, energy	Black hole, selective forwarding, sinkhole, hello flood, wormhole	Limited to attacks covered; no ML integration
ETERS ^11^	Flexible beta distribution trust model	Sybil, black hole, gray hole	CH rotation dependent on energy, may not be optimal in high-mobility scenarios
TaylorC-SSA ^13^	Direct and indirect trust with multi-hop path	Wormhole, hello flood, black hole	Requires higher computation; Taylor expansion can be costly
I-HBA ^15^	Direct, indirect, integrity, forwarding rate	Packet forwarding accuracy, trust level	Complex parameter optimization may slow execution
SEAMHR ^18^	Counter mode cryptography and autoencoder	Secure routing, energy savings	Increased computational complexity
TAOSC-MHR ^19^	Trust with weighted clustering	High latency, low trust nodes, energy efficiency	Limited adaptability to highly mobile networks
DESLR ^20^	Channel reliability, multi-layer trust	Malicious node, packet delay, end-to-end loss	Prone to communication delays in underwater networks
LSR ^21^	Direct and indirect trust for path optimization	Packet delivery, delay minimization	Limited to networks with static nodes
MCRP ^23^	Time ratio threshold for attack detection	Wormhole detection, scalability	Limited adaptability to changing network topology
CM-MH ^25^	Direct trust, indirect trust, encryption	Malicious node filtering, secure path	Limited energy savings in high-security requirements
CTTRG ^27^	Probability-based trust	Black hole, sinkhole, wormhole detection	Computation cost of dynamic trust assessment
Fuzzy-based secure clustering ^29^	Fuzzy logic for trust assessment	Malicious node filtering, delay minimization	Scalability limits due to computational requirements
TACTIRSO ^30^	Exponential moving average trust	Cluster integrity, trust node selection	High dependency on network topology
SLERP ^37^	Chebyshev neural network-based trust	Malicious node detection, energy efficiency	Requires high computational power for ML
CTRF ^34^	Direct, indirect, multi-path trust	Energy-efficient CH selection, secure routing	Computational overhead in high-density networks
Modified ACO with TES ^39^	Trust score, residual energy	CH Selection Optimization, Reliability Assessment	Scalability challenges in dynamic networks
SEaaS with Decentralized Blockchain ^41^	Public key encryption, blockchain verification	Secure data sharing, stakeholder interaction	Potential latency in high-volume transactions
SGM with GTC ^43^	Payoff evaluation, trust-based node selection	QoS integration, secure data forwarding	Overhead in dynamic network conditions
MAGbT Model ^44^	Intrusion detection rate, false positive rate, throughput	Secure packet transmission, node participation assessment	Possible trade-off between accuracy and delay
MAF-DRL ^45^	Trust-based scheduling, agent reliability	Distributed learning, dynamic adaptation to attacks	High energy consumption in multi-agent learning

Existing approaches shows, secure routing in sensor networks often face significant challenges, such as high communication overhead due to reliance on third-party trust evaluations, which can lead to energy inefficiency and network congestion. Machine learning-based techniques require extensive training data, consume excessive energy, and struggle to adapt to dynamic network conditions. Secure routing mechanisms may lack adaptability to multiple attack types and introduce routing delays due to cryptographic overhead. Clustering-based methods frequently result in unequal energy distribution and static CH selection, leading to inefficient routing. Hybrid approaches, while combining multiple strategies, increase complexity and computational overhead. Additionally, many models do not effectively address the dynamic nature of node trust and energy levels, making them vulnerable to malicious activities. This proposed DL-SLnOA approach overcomes these limitations by combining trust-based node selection with SLnO for the identification of CHs, ensuring that only nodes with high trust and energy are chosen. Further it incorporates anomaly behavior detection, where nodes monitor each other’s actions for irregularities that may indicate malicious behavior. This dynamic monitoring helps to quickly identify compromised nodes, enhancing network security by focusing on combined trust, energy levels, and anomaly detection, proposed approach minimizes communication overhead, reduces energy consumption, and provides a more robust, adaptive, and secure solution for routing in sensor networks.

## Proposed system model

**Figure 2:**
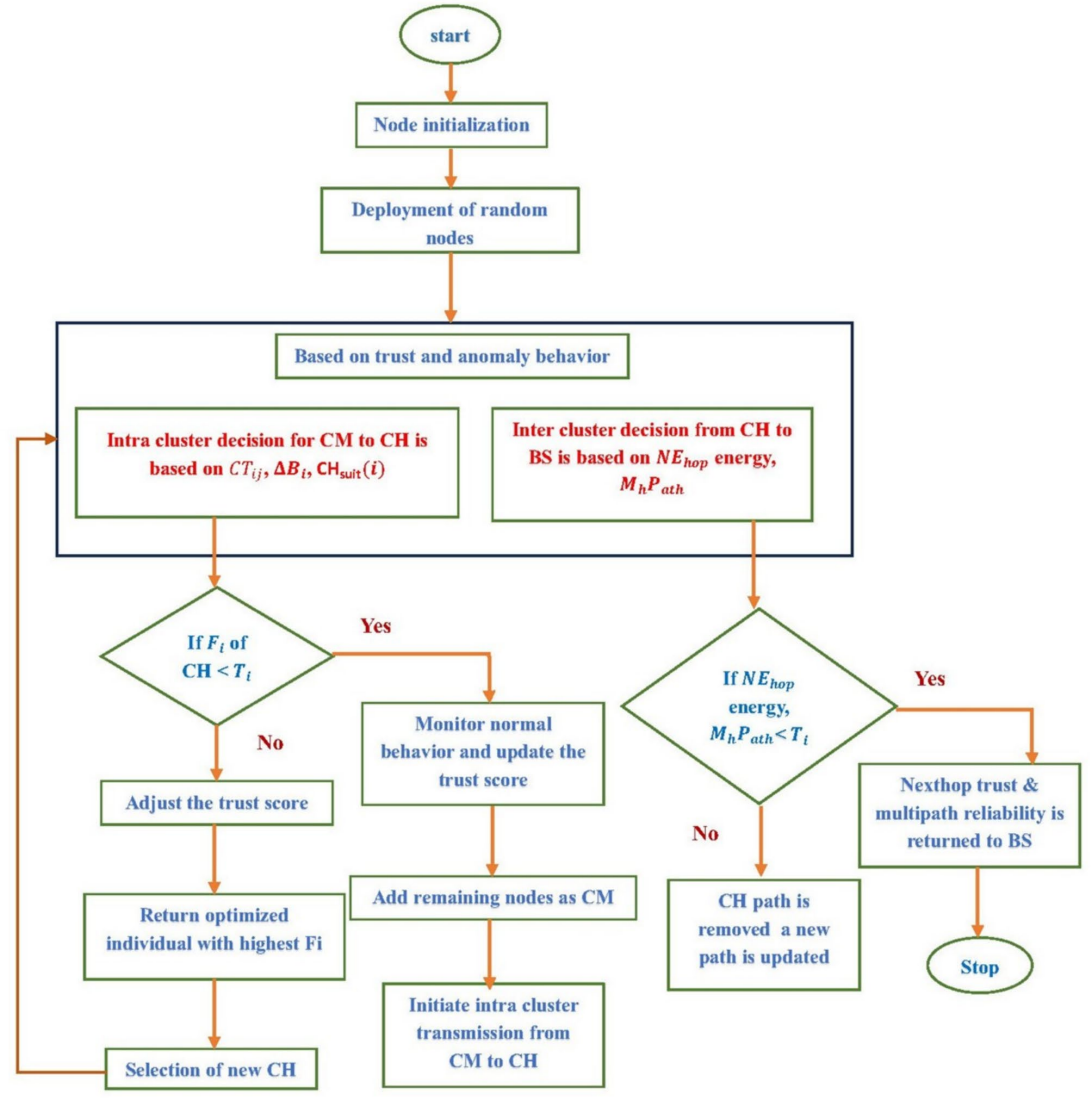
Flow diagram for DL-SLnOA model.

The proposed DL-SLnOA architecture delivers an all-encompassing and flexible architecture for ensuring the secure and energy-efficient operation of WSNs, particularly in mission-critical applications such as border surveillance. Energy imbalance, the early death of nodes, and security threats posed by attackers are the major concerns addressed in this paper. Figure  2 shows the sequence of operations performed in the DL-SLnOA framework. The framework begins with a network start phase in which nodes are randomly deployed within a defined sensing region. The energy values and positional coordinates of the nodes were initially recorded, and the criteria for energy and location-based decisions were established. The clustering process proceeds after the deployment phase, where the network is divided into smaller clusters consisting of sensor nodes. Clustering reduces communication overhead, balances energy consumption, and facilitates data aggregation. In this case, the focus is on selecting CHs; CHs are responsible for data collection and forwarding to the BS. The SLnO algorithm selects CHs considering several parameters, including residual energy, trust level, and distance. In addition, CH locations are dynamically updated following changes in network conditions to avoid the premature depletion of nodes. Routing is further strengthened by the implementation of a dual-layer security system. The first layer focuses on trust-based routing and continuously assesses node behavior, which involves updating and maintaining trust scores based on observed interactions. The second layer focuses on anomaly detection by aiming and isolating misbehaving or malicious nodes that could damage network integrity in any way. Hence, by monitoring behavior and managing trust adaptively, DL-SLnOA can successfully defend nodes from threats such as wormholes and selective forwarding attacks. By applying this routing protocol, only nodes that are considered trustworthy conduct data transmission activities, thereby maintaining data integrity levels. Furthermore, DL-SLnOA is designed to make adaptations to environmental and network conditions respond in real-time to sudden threats or energy drops. These properties make it a weakly centralized and coordinated bioinspired approach. Thus, by integrating security and energy, DL-SLnOA achieves highly scalable and low-overhead coordination among nodes. This dual-layer security method adds a crucial degree of protection and this flexible architecture is the perfect answer for intricate, large-scale deployments since it not only meets the network short-term security and energy efficiency requirements but also fosters its long-term sustainability.

### Network model and energy calculations

The following assumptions about network models are made in order to develop a secure routing system for our dual-layer strategy:

1. Sensor nodes are placed in a random manner around the operational area with primary objective of monitoring and gathering relevant data.
2. It is assumed that each node has equal energy levels, computing power, and storage capabilities. These nodes remain static after deployment, ensuring a consistent and dependable network topology throughout the system operation.
3. A static SN with infinite processing and storage capacity that sits at the network core. SN primary task is receiving and combining data from the sensor nodes dispersed throughout the region.
4. The SN receives all sensor node unique identifiers (IDs) and positional coordinates, which helps it effectively control cluster formation and facilitate network routing.
5. Several clusters of different sizes make up the structure of the sensor network which has Member Nodes (MNs) and CHs. MN is responsible for sensing environmental data and adding energy and trust information about nearby nodes to the data that is gathered are sent to CH. Combining data from MNs, CHs send it to the SN via a multi-hop process through a dual-layer process that combines both SLnO and trust scores. The proposed Algorithm 1 make sure that nodes with high levels of trust and energy are selected as CHs, which lowers the possibility that malicious nodes may assume the position and compromise network security.
6. Once data arrived on SN, each node comprehensive trust score is calculated by combining the received combined trust scores and their residual energy levels, this updated trust information is sent back to network nodes. The revised trust information is then transmitted back to the network nodes by depending on combined trust and energy levels, the network maintains an effective and secure communication process while using the least amount of energy possible. This method does not need any third-party trust evaluation systems, lowering communication overhead and averting possible network congestion. First, a conventional first-order radio model is used to predict the power consumed for each node based on communication with its CH. The energy needed for transmission $E_{\text {tx}}(d_{\text {CH}})$ and reception ${E_{\text {rx}}}$ for a node to communicate with its CH at a distance $d_{\text {CH}}$ is displayed below.

#### Transmission energy $E_{\text {tx}}(d_{\text {CH}})$

Power expenditure for a sensor node to start the transmission and the path-loss model related to signal propagation, the energy needed to send data to its assigned CH varies with distance. A first-order radio model governs the transmission energy $E_{\text {tx}}(d_{\text {CH}})$ needed by a node to communicate with its CH at a distance $d_{\text {CH}}$. In order to assess whether free-space or multi-path propagation effects predominate in the energy loss, this model modifies the transmission energy according to the range between MN and CH, equation  1 provides the definition of the transmission energy. Where, $E_{\text {pow}}$ is the power per bit for transmission/reception, *l* represents the size of data packet which is represented in bits, $\epsilon _{\text {fsp}}$ is a free-space power parameter, $\epsilon _{\text {mpp}}$is a multi-path power parameter,$d_{\text {0}}$ represents threshold distance for free-space and multipath models.

1 $$\begin{aligned} E_{\text {tx}}(d_{\text {CH}}) = {\left\{ \begin{array}{ll} l \cdot E_{\text {pow}} + l \cdot \epsilon _{\text {fsp}} \cdot d_{\text {CH}}^2 & \text {if } d_{\text {CH}} < d_0 \\ l \cdot E_{\text {pow}} + l \cdot \epsilon _{\text {mpp}} \cdot d_{\text {CH}}^4 & \text {if } d_{\text {CH}} \ge d_0 \end{array}\right. } \end{aligned}$$

The free-space model, which requires transmission energy proportional to $d_{\text {CH}}^2$, is more effective at distances smaller than $d_{\text {0}}$. However, multi-path fading takes over and energy consumption scales as $d_{\text {CH}}^4$ when the distance beyond $d_{\text {0}}$. Because it enables nodes to better manage their energy reserves by reducing needless power spending based on closeness to their CHs, this distance-dependent model is essential for WSNs.

#### Reception energy ${E_{\text {rx}}}$

Reception energy ${E_{\text {rx}}}$ usually depends only on the power per bit used during receiving process, it is relatively easier to compute. Since reception power is constant and does not change with distance, the model’s reception energy is unaffected by node’s distance from CH. ${E_{\text {rx}}}$ is given by Eq. (2) where, ${E_{\text {pow}}}$is the power consumed per bit for both transmission and reception.

2 $$\begin{aligned} E_{\text {rx}} = l \cdot E_{\text {pow}} \end{aligned}$$

This equation assumes that receiving data does not involve the same variable energy dissipation as transmission, since the receiver only must power the radio receiver circuitry, regardless of how far away the sender is. This makes the reception energy linear with respect to number of bits received.

#### Total power utilization model

To determine the total power consumed for a node that communicates with its CH, we combine the transmission energy $E_{\text {tx}}(d_{\text {CH}})$ and the reception energy $E_{\text {rx}}$. The total power $E_{\text {total}}$ for every node is given by the sum of these two components, as shown in Eq. (3)

3 $$\begin{aligned} E_{\text {total}} = E_{\text {tx}}(d_{\text {CH}}) + E_{\text {rx}} \end{aligned}$$

By combining the communication and reception energy components, the total power consumption model helps in evaluating the overall energy expenditure of a node in a WSN. This model is essential for making energy-efficient decisions, such as selecting optimal CHs, minimizing communication distance, and balancing the energy usage among nodes.

#### Cluster formation and assignment

Nodes are categorized into clusters depending on their proximity to the CH, minimizing intra-cluster communication distance and energy use. The geometric distance $d_{ij}$ between each node $i$ and $j$ is calculated as each node joins the cluster of the nearest CH based on Eqs. (4) and (5)

4 $$\begin{aligned} d_{ij} = \sqrt{(A_i - A_j)^2 + (B_i - B_j)^2} \end{aligned}$$

5 $$\begin{aligned} CH_i = \arg \min _{j \in \text {CH}} d_{ij} \end{aligned}$$

This assignment reduces the communication distance for each node, minimizing energy consumption and balancing the load across the network.

#### Trust score updating mechanism

In the proposed DL-SLnOA approach, a hybrid trust model is applied to ensure reliable communication and robust node selection. This model combines Direct, Indirect Trust which creates a hybrid trust model called Combined Trust (CoT) to evaluate each node’s reliability.

1. **Direct Trust Score:** Direct trust is determined based on a node’s direct interactions with neighbouring nodes, primarily focusing on packet success rates and energy levels. Equation (6) $DT_{ij}$ metric helps evaluate the node’s reliability and stability.
  6 $$\begin{aligned} DT_{ij} = \alpha \cdot \frac{P_{\text {success}}}{P_{\text {total}}} + \beta \cdot \frac{E_{\text {remaining}}}{E_{\text {initial}}} \end{aligned}$$
  Where $DT_{ij}$ is the direct trust score of node $i$ for node $j$, $P_{\text {success}}$ and $P_{\text {total}}$ represent the successful and total packets sent by node $i$ to node $j$, $E_{\text {remaining}}$ and $E_{\text {initial}}$ are the remaining and initial energy of node $j$, and $\alpha$ and $\beta$ are weights for prioritizing transmission reliability and energy considerations, respectively. The weighting factors $\alpha$ and $\beta$ provide flexibility, allowing adjustments in emphasis on reliability in direct trust evaluation.
2. **Indirect Trust Score:** Indirect trust evaluates the trustworthiness of a node based on recommendations from neighbouring nodes, helping to avoid biases from potentially malicious nodes based on Eq. (7).
  7 $$\begin{aligned} IT_{ij} = \gamma \cdot \frac{\sum _{k \in \text {neighbors}} DT_{ik} \cdot DT_{kj}}{\sum _{k \in \text {neighbors}} DT_{ik}} \end{aligned}$$
  where $IT_{ij}$ represents the indirect trust score of node $j$ as perceived by node $i$, $k$ is a neighbouring node of $i$ that also has a direct trust score for $j$, and $\gamma$ is a scaling factor to balance the influence of the overall score. The indirect trust is calculated by aggregating trust values from multiple neighbors, reducing the impact of any potentially compromised nodes in the trust evaluation.
3. **Combined Trust Score:** The CoT integrates both direct and indirect trust values, with an adjustable weighting factor to emphasize either direct or indirect interactions based on the network’s security requirements.
  8 $$\begin{aligned} CT_{ij} = \lambda \cdot DT_{ij} + (1 - \lambda ) \cdot IT_{ij} \end{aligned}$$
  Equation (8) shows the $CT_{ij}$, Where $CT_{ij}$ is the combined trust score for node $j$ as perceived by node $i$, and $\lambda$ is a weighting factor, typically set between 0.5 and 1 to favor direct trust. This hybrid trust score enables flexible trust management, supporting more reliable and secure node selection within the network by factoring in both observed interactions and neighbouring evaluations. In the proposed DL-SLnOA model, each node maintains a trust score $T_i$, which is updated periodically based on its communication history. The trust score is recalculated at time $t$ as shown in Eq. (9), where $T_i^{(\text {old})}$ is the previous trust score of node $i$, $S_i$ is the success rate of data transmissions, and $\alpha$ is a weighting factor ($0< \alpha < 1$), which allows adjustment between historical and recent behavior:
  9 $$\begin{aligned} T_i^{(\text {new})} = \alpha \cdot T_i^{(\text {old})} + (1 - \alpha ) \cdot S_i \cdot CT_{ij} \end{aligned}$$
  If a node deviates from its expected behavior, its trust score is penalized based on the behavior anomaly metric $\Delta B_i$, calculated as the difference between expected and observed behavior patterns. The updated trust score for node $i$ is shown in Eq. (10), where $\beta$ is the penalty factor for behavior deviation, $\Delta B_i$ is the behavior deviation measure, and $\theta$ is a threshold for detecting behavior anomalies:
  10 $$\begin{aligned} T_i^{(\text {Nnew})} = {\left\{ \begin{array}{ll} T_i^{(\text {new})} - \beta \cdot \Delta B_i&\text {if } \Delta B_i > \theta \end{array}\right. } \end{aligned}$$
  This trust-based security mechanism helps in detecting and isolating malicious nodes, ensuring secure communication in the network.

#### Cluster head suitability score

$CH_{\text {suit}}(i)$ is designed to assess whether a node should remain as a CH based on power, trust, and distance from SN. $CH_{\text {suit}}(i)$ score is calculated as shown in Eq. (11).

11 $$\begin{aligned} CH_{\text {suit}}(i) = {\left\{ \begin{array}{ll} \frac{E_i}{E_{\text {thresh}}} \cdot \frac{T_i}{T_{\text {thresh}}} - \gamma \cdot \frac{D_i}{D_{\text {max}}} & \text {if } E_i \ge E_{\text {thresh}} \text { and } T_i \ge T_{\text {thresh}} \\ \left( \frac{E_i}{E_{\text {thresh}}} \cdot \frac{T_i}{T_{\text {thresh}}} - \gamma \cdot \frac{D_i}{D_{\text {max}}} \right) - \delta & \text {otherwise (rotation)} \end{array}\right. } \ \end{aligned}$$

where, $E_i$ is the power of node $i$,$T_i$ is the trust score of node $i$, $D_i$ is the distance of node $i$ from the sink node (SN) or the cluster center, $E_{\text {thresh}}$ and $T_{\text {thresh}}$ are the minimum energy and trust thresholds required for a node to remain as a cluster head (CH), $D_{\text {max}}$ is the maximum allowed distance from the SN, $\gamma$ is the weighting factor penalizing distance, $\delta$ is the penalty term applied if the node fails to meet the energy or trust thresholds. The suitability score of the node for remaining as a CH is calculated based on energy, trust, and distance from the SN. If the node fails to meet the required thresholds for energy or trust, the suitability score is penalized by $\delta$, leading to a possible rotation of the CH role. Algorithm  1 describes the pseudocode for the CH selection method in the DL-SLnOA model. The computational complexity of SLO will exponentially increase with respect to the network parameters such as number of sensor nodes, node density and network area extensional. When the network population and density increases, the complexity will also increase as the algorithm requires more time to find the optimal solution. In this approach the cluster size is fixed with number of its members, but the number of clusters will get a rise as more number of sensor nodes added to the network. So, the complexity can significantly be controlled and reduced to ensure smoother network operations.

**Algorithm 1:**
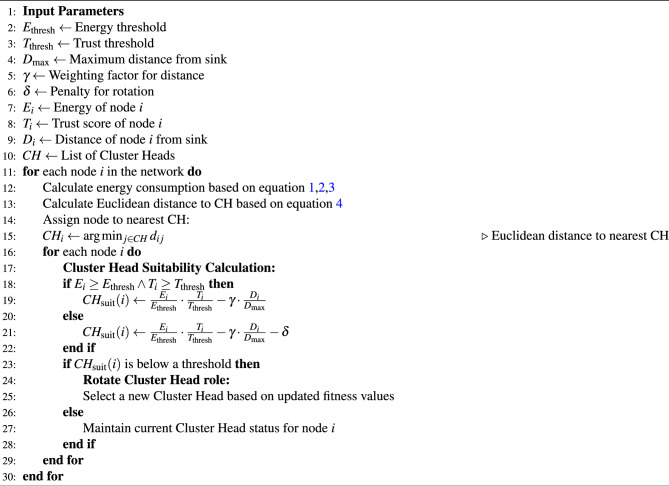
Cluster Head Selection and Rotation

### Clustering-based secure communication routing

The process of clustering in border surveillance plays a crucial role in enhancing communication efficiency, network stability, and security. In DL-SLnOA model optimizing the clustering process by dynamically selecting optimal CHs. The clustering is followed by a multi-hop secure communication routing scheme that integrates trust-based mechanisms to ensure secure data transmission across the network. Algorithm  2 describes the clustering and routing mechanism of DL-SLnOA algorithm. The SLnO algorithm improves clustering by employing an exploration and exploitation mechanism to identify the best CH locations based on node positions.

1. **Exploration Phase:** In the exploration phase, nodes move toward the best CH candidate identified so far, this wide area search space making it more likely to find optimal CH which is centrally located and energy efficient. At iteration *t*, the location of node *i* is updated using Eq. (12). where $\rho$ is the step size that determines how far the node moves towards $T_{\text {best}}$ , $S_{i}^t$ is node position of *i* at iteration *t*, and $T_{\text {best}}$ is the position of the best CH candidate found thus far. This step encourages a more comprehensive network search to find potential CHs, increasing the likelihood of selecting the most centrally located and energy-efficient CHs.
  12 $$\begin{aligned} S_i^{(t+1)} = S_i^t + \rho \cdot (T_{\text {best}} - S_i^t) \end{aligned}$$
2. **Exploitation Phase ** In the exploitation phase of the SLnO algorithm, instead of directly using the fitness value to control CH node rotation, nodes refine their positions based on their proximity to optimal CH location. This movement is guided by using Eq. (13), where $S_i^{(t+1)}$ is the updated node position *i*, $S_i^{t}$ is the current position, $T_{\text {best}}$ is the best CH candidate, $D_i$ is the spatial distance between nodes *i* and $T_{\text {best}}$ , and $D_{\text {max}}$ is the maximum distance in the network. The factor $\left( 1 - \frac{D_i}{D_{\text {max}}} \right)$ decreases as nodes get closer to the best solution to ensure gradual convergence, while the parameter $\lambda$ controls the step size.
  13 $$\begin{aligned} S_i^{(t+1)} = S_i^t + \lambda \cdot (T_{\text {best}} - S_i^t) \cdot \left( 1 - \frac{D_i}{D_{\text {max}}} \right) \end{aligned}$$

**Algorithm 2:**
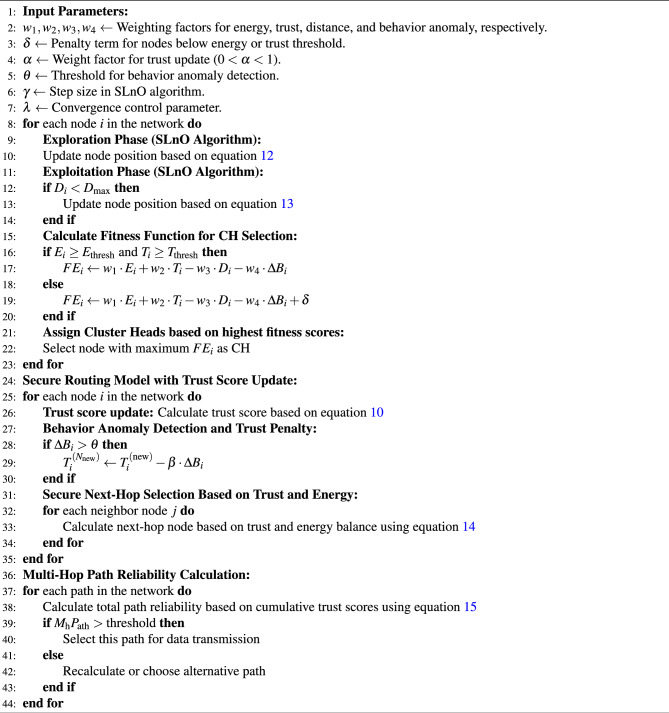
DL-SLnOA based Clustering and Routing

#### Routing and secure communication

After selecting CHs, data is gathered by the nodes and transmitted to the SN through the selected CHs. A multi-hop routing model is used to relay data when direct communication is not possible. This routing model ensures that the network remains operational even when nodes are too far from the SN, optimizing network lifetime and communication efficiency. To secure the routing process, a secure next hop selection and multihop path reliability model is implemented, which includes trust score updates and behaviour anomaly detection mechanisms to ensure secure communication. This is crucial in a border surveillance setting, where malicious nodes can disrupt network functionality.

#### Trust and residual power based secure next-hop selection

Next-Hop (NE_hop) selection process in a routing protocol is crucial for ensuring that data packets are forwarded through reliable and energy-efficient nodes. Selecting the next node for forwarding data is a decision that impacts the network’s security, efficiency, and longevity.

14 $$\begin{aligned} \text {NE\_hop} = \arg \max _{j \in \text {neighbors}} \left( \lambda \cdot CT_{ij} + (1 - \lambda ) \cdot \frac{E_{\text {remaining}}}{E_{\text {initial}}} \right) \end{aligned}$$

In Eq. (14), each node chooses its $\text {NE\_hop}$ node (for forwarding data) based on a balance of trust score $CT_{ij}$ and energy levels. Here, $\frac{E_{\text {remaining}}}{E_{\text {initial}}}$ is the ratio of remaining and initial power for node $j$, representing its residual energy status. $\lambda$ is a weighting factor that can be adjusted based on network priorities (e.g., if reliability is prioritized, $\lambda$ is higher; if energy conservation is key, $\lambda$ is lower). This approach ensures that only nodes with both high trustworthiness and sufficient energy are selected as next-hops, which enhances both security and the longevity of the network.

#### Multi-hop path reliability

In a multi-hop network, data packets typically pass through several intermediate nodes before reaching their destination. The reliability of the entire path depends on the reliability of each node along the route by evaluating the trustworthiness of nodes in a path, the network can determine how secure and stable the communication.

15 $$\begin{aligned} M_h P_{\text {ath}} = \sum _{k \in \text {path}} CT_{ik} \end{aligned}$$

The total reliability of a path $M_h P_{\text {ath}}$ is computed as the sum of combined trust scores $CT_{ik}$ for each node $k$ along the path. Higher cumulative scores represent paths that are more reliable and resistant to threats from malicious nodes by calculating path reliability, the network can avoid routes that may be compromised by low-trust nodes. Equation 15 helps the routing protocol select paths with maximum trustworthiness, mitigating the risks associated with forwarding data through potentially malicious nodes.

#### Fitness evaluation for selection and routing

**Fitness evaluation for choosing the routing paths** incorporates the updated trust score and secure routing parameters, ensuring that nodes with high energy, reliable communication, and minimal behavior anomalies are prioritized. The fitness evaluation $FE_i$ for each node $i$ is shown in equation 16.

16 $$\begin{aligned} FE_i = {\left\{ \begin{array}{ll} w_1 \cdot E_i + w_2 \cdot T_i - w_3 \cdot D_i - w_4 \cdot \Delta B_i & \text {if } E_i \ge E_{\text {thresh}} \text { and } T_i \ge T_{\text {thresh}} \\ w_1 \cdot E_i + w_2 \cdot T_i - w_3 \cdot D_i - w_4 \cdot \Delta B_i + \delta & \text {otherwise (rotation)} \end{array}\right. } \end{aligned}$$

where, $w_1, w_2, w_3, w_4$ are weighting factors for energy $E_i$, trust $T_i$, distance $D_i$, and behavior anomaly metric $\Delta B_i$, and $\delta$ is a penalty term applied to nodes that do not meet the energy or trust thresholds. The behavior anomaly term $\Delta B_i$ helps identify nodes exhibiting abnormal activity, further refining the routing decisions. Nodes with high behavior deviations will have their fitness score reduced, thus discouraging their selection as CH in the routing path.Since sensor nodes are statically placed with no mobility feature, a node or link failure possibility can be avoided with balanced workload assignment to all the nodes in the network. The limitation of this study could be the fact that not addressing route failure and link reliability parameters.

### Computational complexity analysis

In dense network complexity grows quadratically based on the number of connections in each layer, yet it is a straight-forward to implement and is effective as well in classification with long training time and high memory. Depending on the scalability, the computation cost and overhead differs. In sparse networks, complexity grows linearly with number of nodes in WSN that achieves reduced memory usage and computation time but difficult to implement due to the fact of sparsity handling requires more sophisticated techniques to train and optimize WSN. In our proposed model, the complexity is average as the medium sized WSN is deployed to evaluate the model’s performance. When the network size become large scale, the hardware and software parameters needs to be advanced. The computational complexity of the proposed DL-SLnOA approach is primarily determined by two factors: the SLnO-based clustering process and the secure routing mechanism. The clustering process involves the SLnO algorithm, where each of the $N$ nodes updates its position over $I$ iterations to optimize the selection of CHs. Thus, the time complexity for the SLnO clustering phase is $O(N \times I)$. For the secure routing phase, next-hop selection involves a relatively simple process where each node communicates only with its CH, which results in a linear time complexity with respect to the quantity of nodes. Hence, the routing complexity is $O(N)$. The multipath evaluation is optimized using a greedy approach and path pruning, with a time complexity of $O(N \times d)$, where $d$ is the average neighbor count per node. The overall computational complexity of the proposed approach is given in Eq. (17)

17 $$\begin{aligned} O(N \times I + N \times d) \end{aligned}$$

This shows that the model is efficient and scalable, as the iteration count $I$ is typically small and $d$, the average neighbor count, is often much smaller than $N$. Therefore, the overall complexity primarily scales linearly with the number of nodes $N$, making it suitable for large-scale WSNs used in applications like border surveillance.

## Simulation setup

This section discusses the performance of the proposed dual-layer approach for detecting malignant nodes is evaluated. This proposed approach integrates two key strategies: SLnO and trust score with anomaly behaviour detection to detect and neutralize attacks, such as WHA and SFA. The network architecture is setup in MATLAB 2021a simulation environment with windows 11 and Intel core 5 processor which includes 500 static, homogeneous nodes randomly deployed within a 1000$\times$1000meter area, with a SN positioned at (200,500) of sensing area and other parameters are shown in Table  2. Comparison is made between the proposed method and other well-known approaches, including TAOSC-MHR  ^18^, LSR  ^20^, EERRR  ^16^, and EMBTR  ^31^ to assess performance across various key metrics namely packet transmission efficiency, speed of malicious node detection and network energy utilization. The validation of proposed model in comparison with other models were not performed, but either confidence intervals or standard deviation would likely be considered to validate the model results in our future work or enhancing the current model further.

**Table 2:** Key parameters for simulation setup and configuration.

Parameters	Values
Sensing area	1000 $\times$ 1000 m
Number of sensor nodes	100–500
Base station location	(200, 500)
Initial energy per node	1 J
Data payload size	1000 bits
Network control packet size	200 bits
Communication range per node	30 m
Maximum iterations	500
Proportion of malicious nodes	10%
Idle state power consumption	$0.06 nJ/bit/m^{2}$
Active mode power consumption	$0.0175 nJ/bit/m^{2}$
Transmission power consumption	$0.744 nJ/bit/m^{2}$
Receiving power consumption	$0.0648 nJ/bit/m^{2}$
Amplifier power consumption	$0.0013 nJ/bit/m^{2}$

Assessing effectiveness and efficiency of proposed DL-SLnOA dual-layer approach for detecting malignant nodes in the network, several key performance indicators are defined. These criteria are employed to assess the overall security, power efficiency, and reliability under various attack scenarios, including WHA and SFA. The DL-SLnOA which integrates SLnO, trust score calculations, and anomaly behaviour detection, aims to enhance the detection speed, minimize energy consumption, and maintain high packet transmission efficiency. The comprehensive trust value is crucial for ensuring the network’s reliability and security. Nodes with a trust value above a predefined threshold are considered trustworthy. The proposed DL-SLnOA approach combines the trust score and the anomaly behaviour detection module to identify malicious nodes in the network quickly in wormhole and selective forwarding attacks, the trust scores decrease progressively depending on their behaviours. Malicious nodes in wormhole attack manipulate the network routing by forwarding packets over long distances. In selective forwarding attack malicious nodes drop packets selectively instead of forwarding them, leading to packet loss and compromised communication.

### Speed of malignant node detection

**Figure 3:**
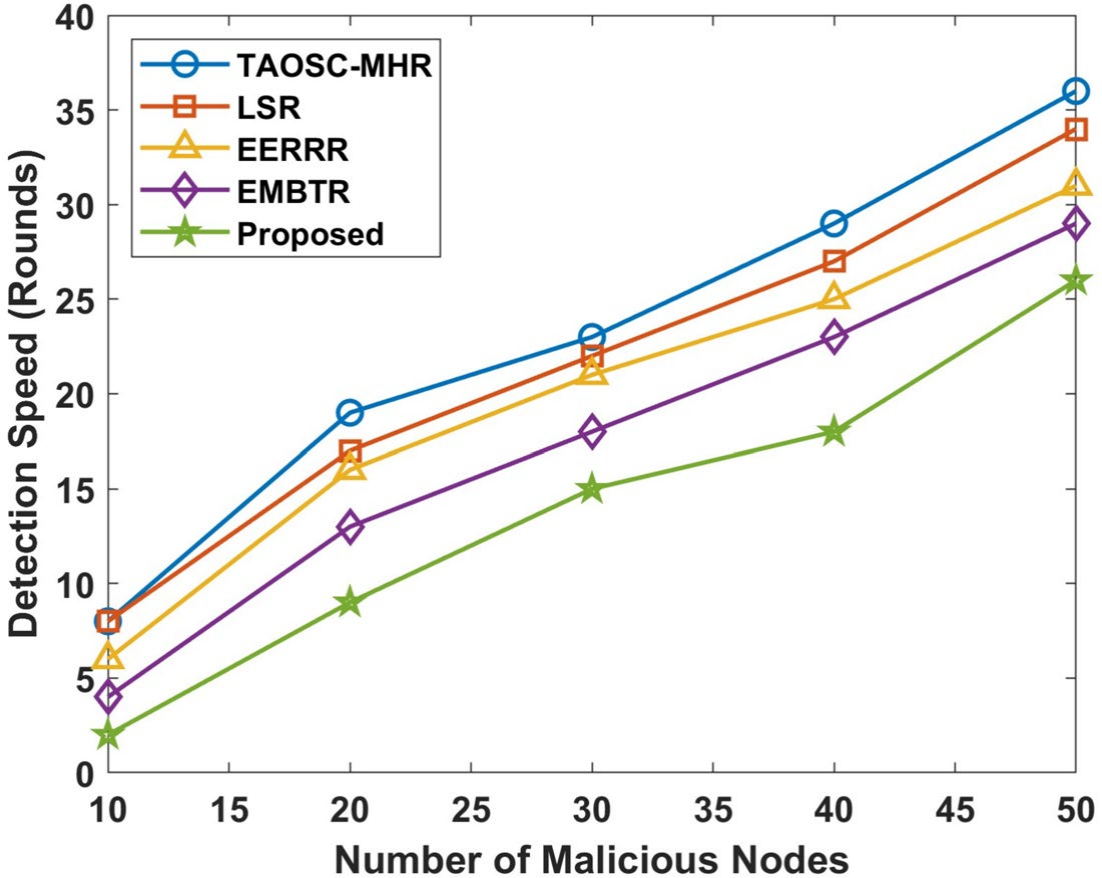
Speed of malicious node detection in Worm hole attack.

**Figure 4:**
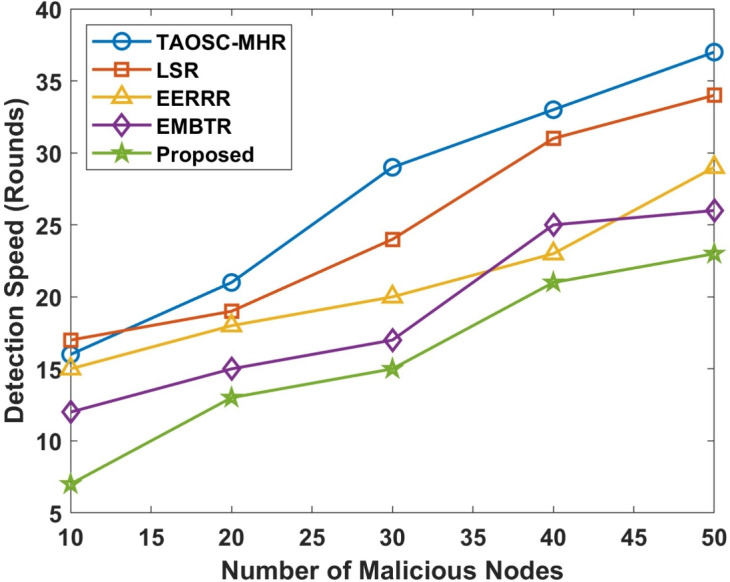
Speed of malicious node detection in selective forwarding attack.

The malignant node detection speed is a crucial metric for minimizing unnecessary packet loss and improving overall network performance. Tables 3, 4 and Figs. 3, 4 depicts the speed of malicious node of the proposed model compared with other approaches.

**Table 3:** Comparison for speed of malicious node detection in Wormhole attack.

Malicious nodes	TAOSC-MHR	LSR	EERRR	EMBTR	Proposed
10	8	8	6	4	2
20	19	17	16	13	9
30	23	22	21	18	15
40	29	27	25	23	18
50	36	34	31	29	23

**Table 4:** Comparison for speed of malicious node detection in selective forwarding attack.

Malicious nodes	TAOSC-MHR	LSR	EERRR	EMBTR	Proposed
10	16	17	15	12	7
20	21	19	18	15	13
30	29	24	20	17	15
40	33	31	23	25	21
50	37	34	29	26	25

The proposed DL-SLnOA approach, leveraging both SLnO and anomaly detection, provides a faster response to 50 malicious node activities compared to other approaches. In wormhole attack the DL-SLnOA approach detected malicious nodes in 23 rounds, while TAOSC-MHR, LSR, EERRR, and EMBTR took 36, 34,31, and 29 rounds respectively. Selective forwarding attack the detection speed for this attack was also faster with the proposed approach, taking only 25 rounds to detect malicious nodes, compared to 37 rounds for TAOSC-MHR, 34 rounds for LSR, 29 rounds for EERRR, and 26 rounds for EMBTR. The integration of SLnO and anomaly behaviour detection allows for adaptive penalties and historical value tracking, significantly speeding up malicious node identification.

#### Packet transmission efficiency

The Packet transmission efficiency (PTE) is a key metric that reflects the overall network performance, particularly in terms of how well the network handles packet forwarding in the presence of attacks. Higher PTE signifies the network can transfer packets with minimal loss, despite the existence of malignant nodes. In wormhole attack as malignant nodes are introduced within a network as PTE decreases.

**Table 5:** Comparison of efficient packet transmission in selective forwarding attack.

Malicious nodes	TAOSC-MHR	LSR	EERRR	EMBTR	Proposed
10	0.949	0.950	0.954	0.971	0.979
20	0.915	0.922	0.926	0.940	0.955
30	0.881	0.910	0.914	0.927	0.942
40	0.875	0.899	0.901	0.914	0.930
50	0.830	0.851	0.872	0.884	0.897

**Figure 5:**
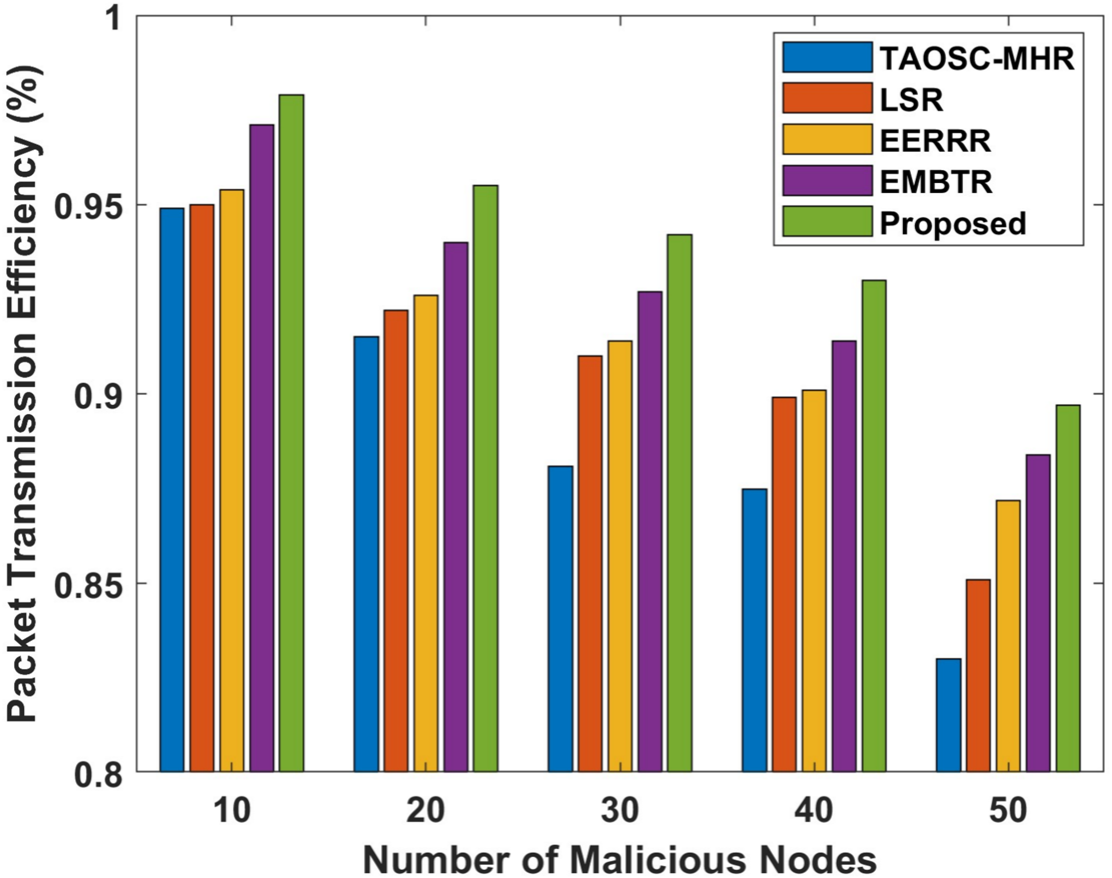
Packet transmission efficiency in wormhole attack.

**Table 6:** Comparison of efficient packet transmission in wormhole attack.

Malicious nodes	TAOSC-MHR	LSR	EERRR	EMBTR	Proposed
10	0.953	0.961	0.960	0.974	0.983
20	0.914	0.924	0.920	0.966	0.974
30	0.899	0.915	0.901	0.941	0.966
40	0.862	0.878	0.882	0.910	0.923
50	0.832	0.853	0.872	0.884	0.905

**Figure 6:**
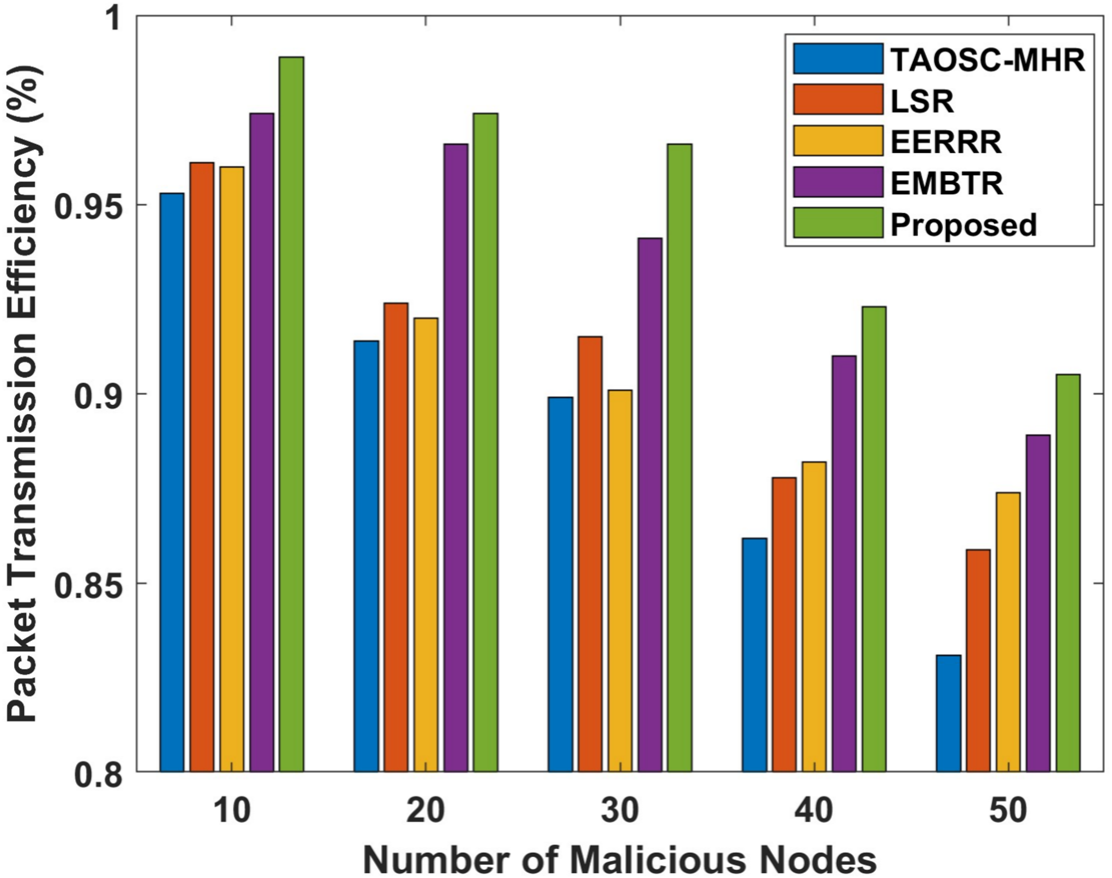
Packet transmission efficiency in selective forwarding attack.

Tables 5, 6 and Figs. 5, 6 depicts the packet transmission efficiency of the proposed model compared with other approaches. The proposed DL-SLnOA approach manages to maintain a higher PTE compared to the other approaches. The DL-SLnOA approach maintained a PTE of 89.7%, compared to TAOSC-MHR 83%, LSR 85.1%, EERRR 87.2%, and EMBTR 88.4%. In selective forwarding attack were malicious nodes selectively drop packets; the PTE decreases more significantly. Despite this, the DL-SLnOA approach still outperformed the others with a PTE of 90.5%, outperforming TAOSC-MHR 83.2%, LSR 85.3%, EERRR 87.2%, and EMBTR 88.4%.

#### Network energy utilization

Energy consumption is a critical metric, as energy efficiency directly affects the network’s lifetime.

**Table 7:** Comparison of average energy utilization with varying nodes density.

Nodes	TAOSC-MHR	LSR	EERRR	EMBTR	Proposed
100	0.0160	0.0157	0.0151	0.0138	0.0122
200	0.0163	0.0171	0.0164	0.0144	0.0130
300	0.0184	0.0186	0.0179	0.0151	0.0141
400	0.0199	0.0194	0.0195	0.0154	0.0143
500	0.0230	0.0211	0.0200	0.0162	0.0155

**Figure 7:**
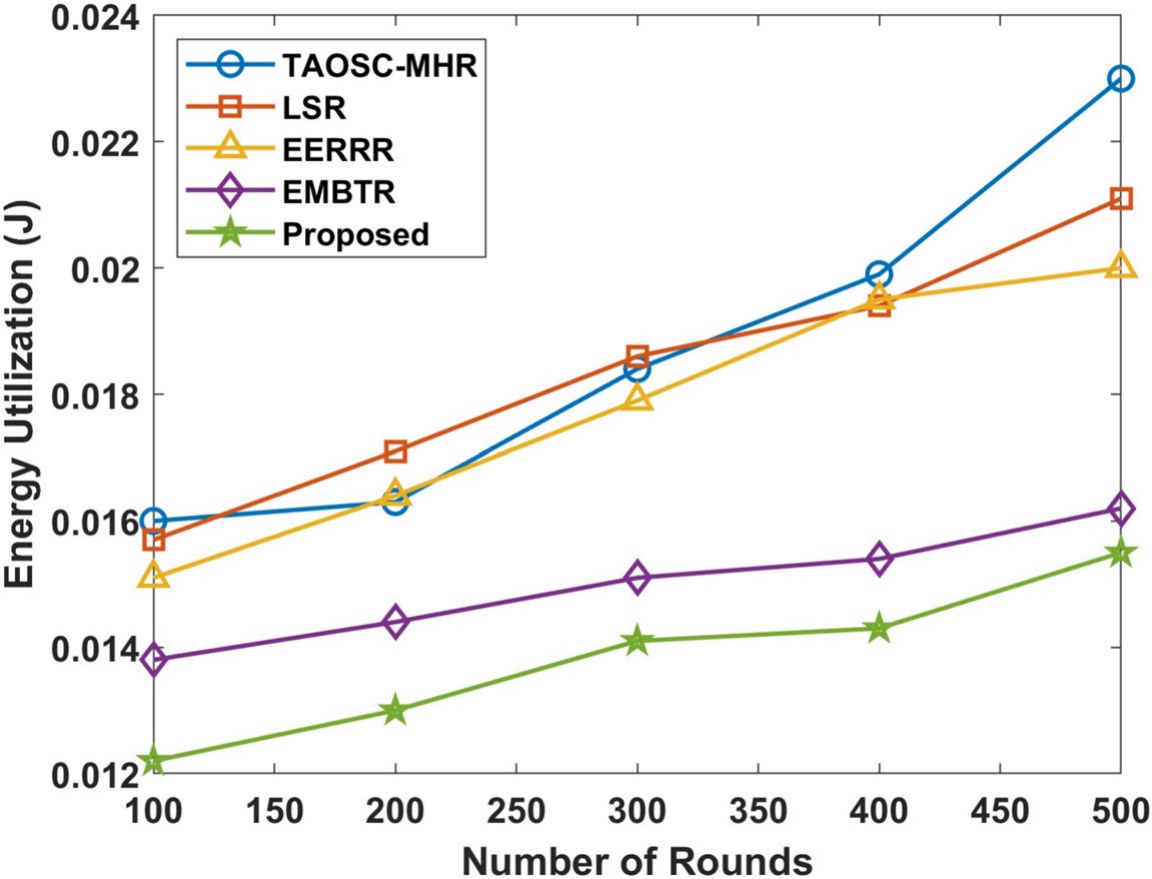
Average network energy utilization.

Table  7 and Fig. 7 depicts the network energy utilization of the proposed model compared with other approaches. The proposed DL-SLnOA approach aims to minimize energy consumption by selecting energy-efficient nodes as CH by integrating SLnO and trust-based decision-making, this approach ensures that nodes with higher trust scores and residual energy are selected for communication tasks, thereby reducing overall energy consumption. Energy consumption increases over the course of the simulation, especially as more rounds are conducted. However, the DL-SLnOA approach utilized 0.0155J which outperforms other techniques, such as 0.230J for TAOSC-MHR, 0.0211J for LSR, 0.020J for EERRR, and 0.0162J for EMBTR. This adaptive trust mechanism and SLnO algorithm helped to reduce unnecessary energy dissipation by optimally selecting routes and CH.

#### Detection accuracy

The detection of malicious node intruding into the WSN network area is measured by three metric values, namely, True Positive Rate (TPR), False Positive Rate (FPR) and f1-score. In this work, numerous models were assessed against their detection accuracy when applied to SFA and WHA.

**Figure 8:**
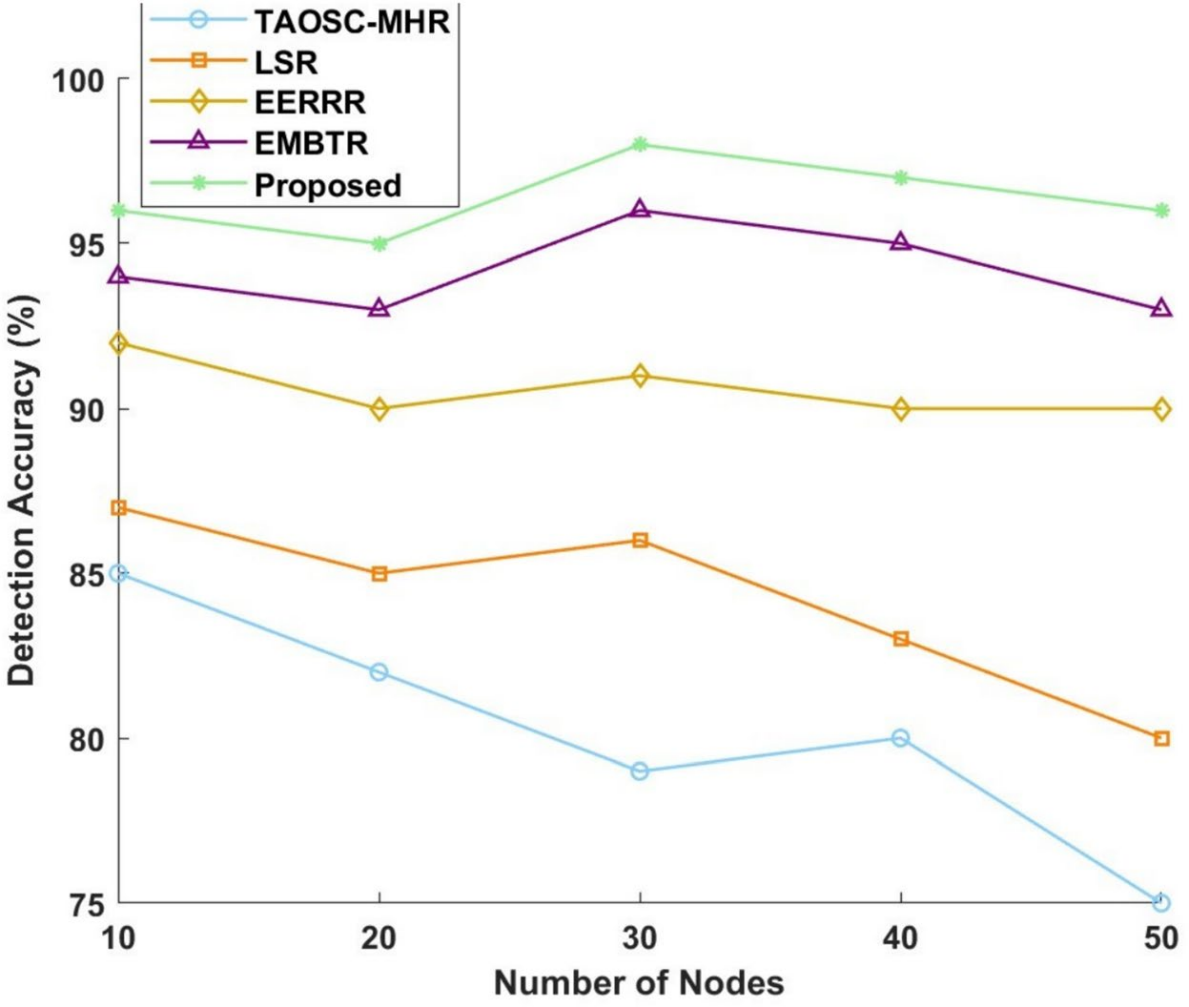
Detection accuracy for selective forwarding attack.

**Figure 9:**
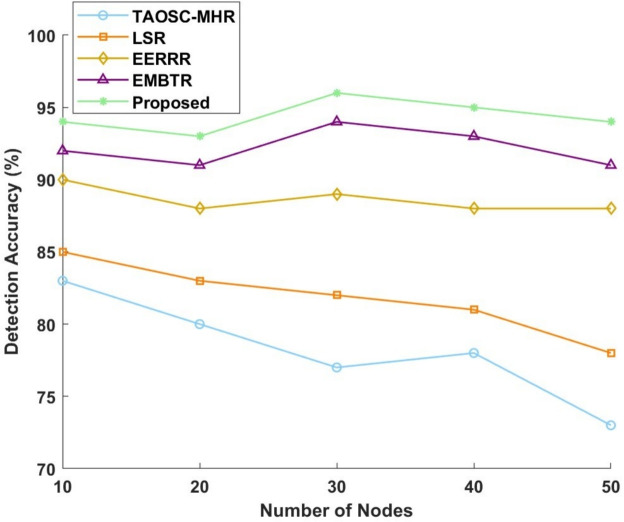
Detection accuracy for worm hole attack.

Figures 8 and 9 presents a graphical view of the performance metrics. The proposed model always beat all other models to secure the highest accuracy for all node counts. The proposed model performance curve is for SFA starting at 96% for 10 nodes, remaining at 95% for 20 nodes, acquiring a maximum value of 98% for 30 nodes, and, grouped in 40 and 50 nodes against 97 and 96%, respectively. Conversely, in a bitterly competitive way, TAOSC-MHR dropped farthest from a good score of 85% on detection for the 10-node case to 75% for 50. Both LSR and EERRR, meanwhile, fluctuated less, with LSR starting hits of 87% at 10 nodes, falling to 80% at 50 nodes, and EERRR starting at 92% for 10 nodes and from there oscillating between 90% and 92% for the 50 nodes. Likewise, EMBTR ranked second, starting performances at 94% for 10 nodes, peaking at 96% for 30 nodes, and thereafter dipping slightly to 93% for 50 nodes. In the case of wormhole attack is concerned, detection performance was maintained at a high of 94% for 10 nodes by the proposed model, thus rising to 96% for 30 nodes and stabilizing at 95% for 50 nodes. With TAOSC-MHR, we observe a decline in detection accuracy from 83% for 10 nodes all the way down to 73% for 50 nodes. Meanwhile, LSR gives an overall downward trend from 85% for 10 nodes to 78% for 50 nodes. EERRR can start with a detection rate of about 90% with 10 nodes and stabilizes from 88% to 90% until 50 nodes. EMBTR reports 92% with 10 nodes, attains the zenith of 94% for 30 nodes, and drops to 91% detection rate at 50 nodes. Thus high in performance, the proposed model adds to the utmost detection accuracy for both attacks, massively surpassing the other models.

#### False positive rate

**Figure 10:**
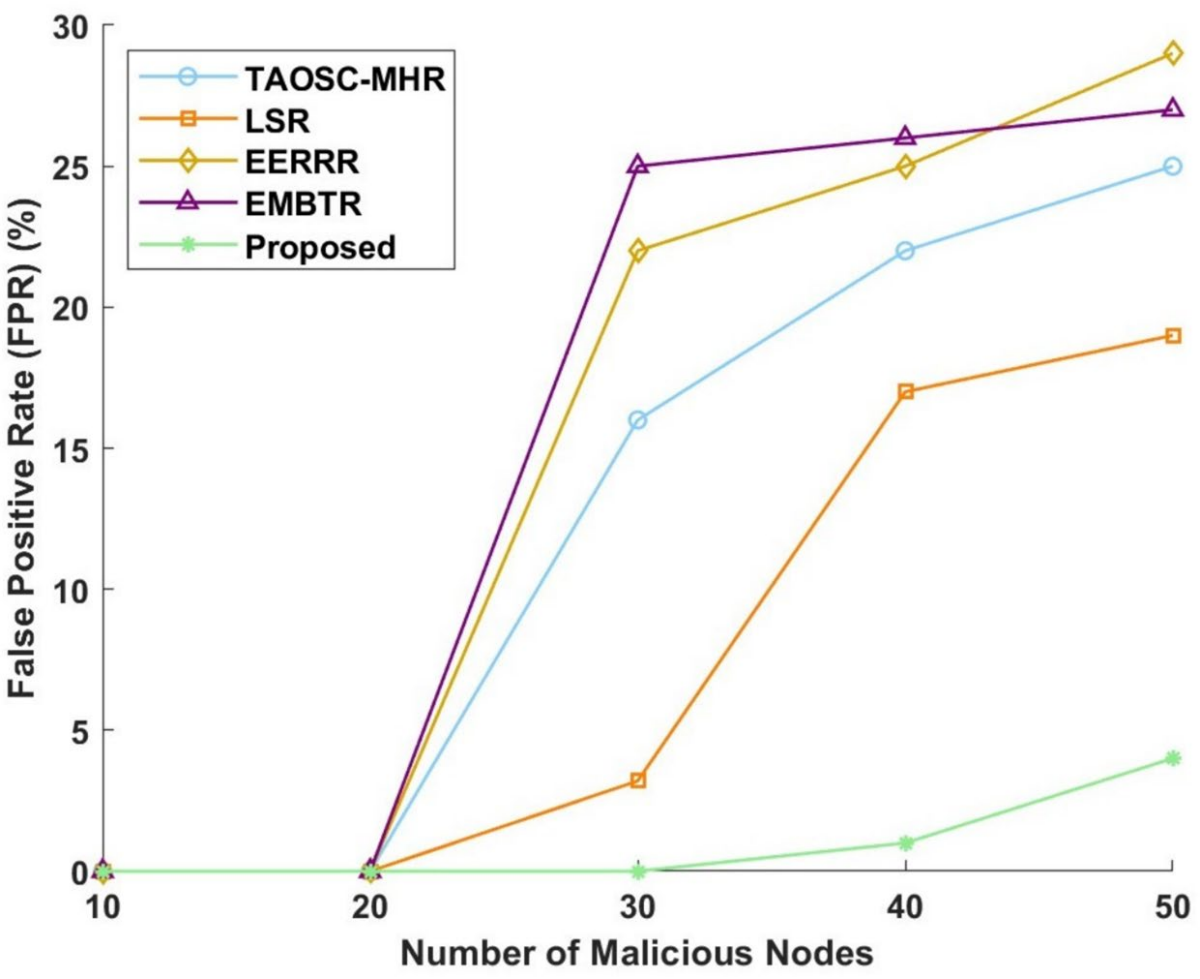
False positive rate for selective forwarding attack.

**Figure 11:**
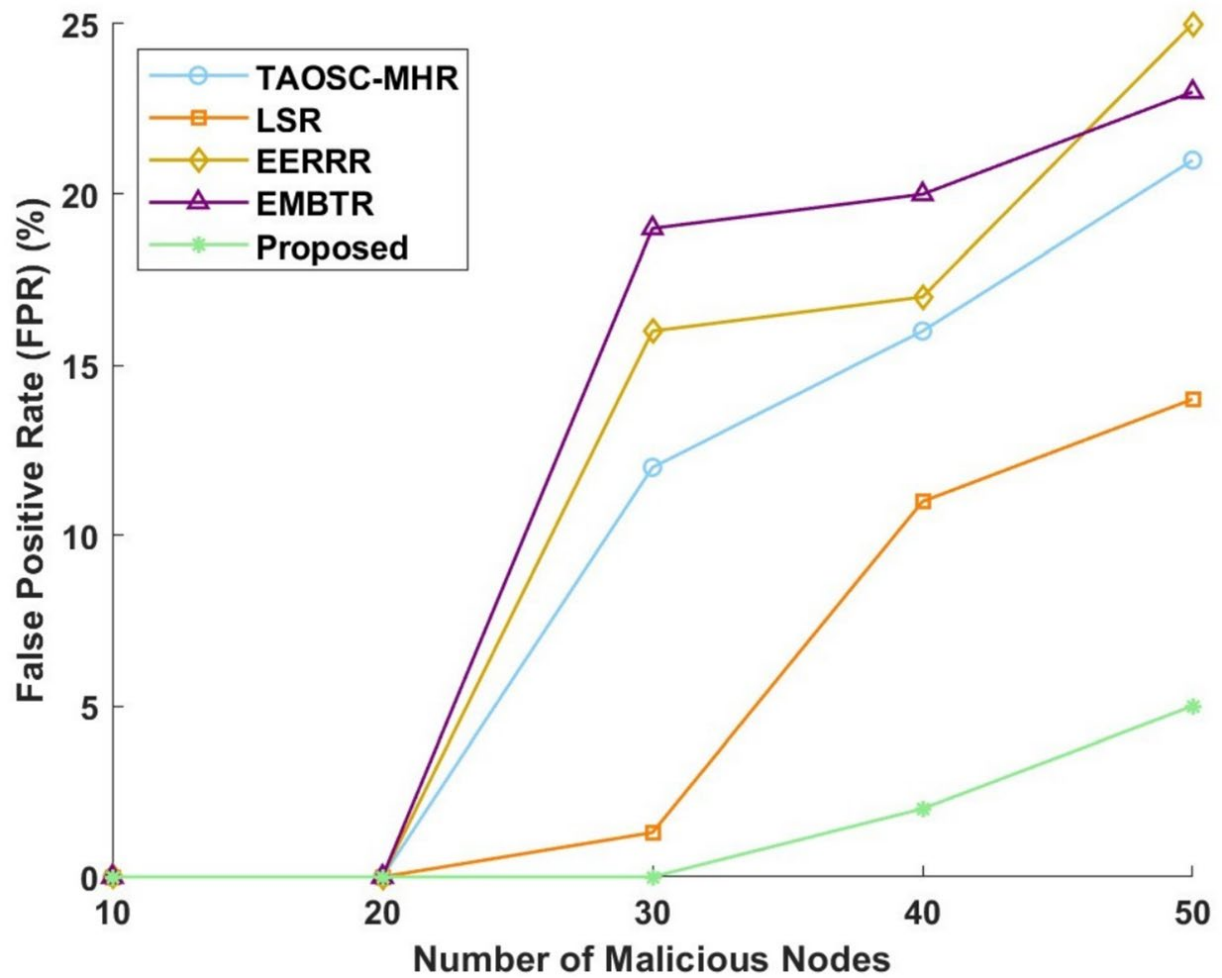
False positive rate for worm hole attack.

False positive rates or FPRs of the various models for SFA and WHA are shown in Figs. 10 and 11. The proposed model has achieved a very low FPR thereby showing its effectiveness in the reduction of false detections. In terms of SFA for which the proposed model maintains 0% FPR for 10 and 20 nodes, slightly increases to 1% at 40 nodes and to 2% for 50 nodes. Meanwhile, TAOSC-MHR follows a continuing increasing pattern from 16% for 30 nodes, up to 22% for 40 nodes, and finally 25% for 50 nodes. Meanwhile, LSR is also increasing upward to obtain 3.2% for 30 nodes, 17% for 40 nodes, and 19% for 50 nodes. EERRR remains low in FPR values up to 20 nodes but later increases to 22% at 30 nodes, 25% at 40 nodes, and 29% at 50 nodes. EMBTR had the highest FPR, with FPR values reaching 25% for 30 nodes, peaking at 26% for 40 nodes, and pretty much maintaining at 27% for 50 nodes.

In the case of WHA, the proposed model maintains an FPR of 0% for both 10 as well as 20 nodes, slightly increasing itself to 2% with increase to 40 nodes and 7% with increase to 50 nodes. TAOSC-MHR started from 12% for 30 nodes before finally increasing to 16% and 21% for 40 and 50 nodes consecutively. LSR has a value of 3% for 30 nodes, 11% for 40 nodes, and 14% for 50 nodes. EERRR has 0% from 0 to 20 nodes and later increases to 16% for 30 nodes, 17% for 40 nodes, and 25% for 50 nodes. EMBTR also carries the highest FPR in this regard as well wherein it starts from 19% for 30 nodes, increases to 20% for 40 nodes, and then at 23% for 50 nodes. Hence from these results, the conclusion can be arrived at that the new model has the lowest False Positive Rate in all attack scenarios thus favoring it as a more leak-proof detection approach compared to the earlier ones.

## Conclusion and opportunities of future research

This study introduces a robust and adaptive clustering and routing model tailored for WSNs, with a particular emphasis on critical security applications like border surveillance through the innovative integration of the SLnO algorithm. The DL-SLnOA model effectively balances the energy efficiency and trustworthiness of nodes, ensuring that CH selection dynamically adapts to the network’s ongoing operational demands by using exploration and exploitation stages and determines the best locations for CHs based on node proximity, energy availability, and communication efficiency, which significantly lowers redundant energy use, this dual-layer security approach incorporates real-time behaviour anomaly detection and trust score updates, also results in a strong defence against malicious attacks. This preserves data integrity and improves network dependability in hostile or high-risk locations by reducing energy consumption, it not only extends the network lifespan but also increases its resistance to node malfunctions, ensuring steady performance in a range of scenarios. As compared to other models, proposed approach shows superior detection accuracy while dramatically decreasing the rate of false positives, thereby giving great reliability in identifying malicious nodes. It further reduces the misclassification error compared to certain existing ones, thereby enhancing the security of the network. Thus, DL-SLnOA model presents a scalable, secure, and highly proficient solution for sustained WSN installations, demonstrating its applicability for a range of mission-critical scenarios where reliable, secure monitoring is essential. Future enhancements to the proposed model could focus on integrating advanced techniques to further improve network performance and security. Implementing AI-based anomaly detection with our already existing model would enhance the identification step of malicious nodes while at the same time reduce the false positives. Future works on machine learning might also include parameter activation and adaptation for SLnO to optimize online performance under real-time conditions and improve adaptability to changing network conditions. In surveillance scenarios with these kinds of nodes, the integration could potentially enhance the coverage area, while energy harvesting techniques would help in achieving sustainable network operations. QoS-aware routing would offer reliable communication for mission-critical applications, which is indispensable for real-time data transmission. Further research can also focus on integrating multi-tier clustering strategies to allow scalability and strong resiliency in the DL-SLnOA model, which is important when deploying an efficient large-scale deployment in extensive regions.

## Data Availability

All data analyzed during this study are included in this article.
